# Quarterly Summary of Transactions of the College of Physicians of Philadelphia

**Published:** 1860-11

**Authors:** 


ART. XIII.—*Quarterly Summary of Transactions of the College of Physicians of Philadelphia.* Philadelphia, 1860.

THE most interesting article, perhaps, in the present summary, is that by Dr. Da Costa, on the "Effect of Respiration on the size of the Heart," founded upon experimental investigation; but as this will be analyzed in our abstracts, no further notice need be taken of it here.

The other articles are, on arsenic in chronic bronchitis, and on stomatorrhœa vicarious of the menses, by Dr. Wood; on common salt in epistaxis, by Dr. Hays; ligature of the right subclavian artery for axillary aneurism, by Dr. H. E. Drayton; chronic inflammation and softening of the brain, and emphysema, with deep-seated tubercle of the lung, by Dr. Levick; congenital fissure of the upper half of the sternum, by Dr. A. M. Slocum; imperforate rectum, by Dr. Ellerslie Wallace; and, finally, the report on meteorology and epidemics for 1859, by Dr. Wilson Jewell. This report is a very elaborate and instructive one, reflecting great credit upon the talents and industry of the distinguished author. We have room only for the following extract, from which it will be perceived that the mortality of Philadelphia, in comparison with that of some of the other principal cities of the Union, is remarkably small:—

"A comparison of the rate of mortality to population of our own with several other cities, will show still more clearly the striking difference in the death-rate pressure upon populations in different places.

| | Population. | Mortality. | Ratio of deaths to pop. | Deaths to each 1,000. | Per cent. of deaths to pop. |
|---|---|---|---|---|---|
| Providence | 52,000 | 982 | 1 in 52·09 | 19 | 1·83 |
| Boston | 180,000 | 3,738 | 1 in 48·15 | 21 | 2·07 |
| New York | 800,000 | 21,645 | 1 in 36·09 | 27 | 2·70 |
| Philadelphia | 625,000 | 9,742 | 1 in 64·15 | 15 | 1·55 |
| Baltimore | 253,000 | 5,039 | 1 in 50·02 | 20 | 2· |

The remaining portions of the Summary are occupied with the memoirs of Dr. Thomas H. Yardley and Dr. Henry Bond, two members of the College, who died during the past and present years; the first being from the pen of Dr. Littell, and the other from that of Dr. Condie. Both these gentlemen were well known in this city, and were universally respected for their professional attainments and the integrity of their character.

Dr. Yardley was a native of Wakefield, Bucks County, Pennsylvania, and at the time of his death had just entered upon his sixtieth year. A private pupil of the late Dr. Joseph Parrish, he took his medical degree in the University of Pennsylvania in 1825, and was for many years in the enjoyment of a large and lucrative practice. The immediate cause of his lamented death was disease of the heart.

Dr. Bond, born at Watertown, Massachusetts, in 1790, died of apoplexy at the age of sixty nine. His name is widely known in this country in association with Bond's splints for fractures of the radius, Bond's œsophagus forceps, and other instruments and apparatus. In 1856, he published two octavo volumes of upwards of twelve hundred pages, entitled "Watertown Family Memorials," with notes and illustrations. He also occasionally contributed an article to the periodical press. He has left an unpublished manuscript, exhibiting, in tabular form, the statistics of 1307 cases of labor. He bequeathed a portion of his library to the College of Physicians, and the bulk of his estate, after the death of some of his heirs, to Dartmouth College. He had never been married.

